# Cerebrospinal fluid cytotoxicity in amyotrophic lateral sclerosis: a systematic review of *in vitro* studies

**DOI:** 10.1093/braincomms/fcaa121

**Published:** 2020-08-06

**Authors:** Koy Chong Ng Kee Kwong, Jenna M Gregory, Suvankar Pal, Siddharthan Chandran, Arpan R Mehta

**Affiliations:** f1 Centre for Clinical Brain Sciences, University of Edinburgh, Edinburgh, UK; f2 UK Dementia Research Institute at University of Edinburgh, Edinburgh, UK; f3 Euan MacDonald Centre for MND Research, University of Edinburgh, Edinburgh, UK; f4 MRC Edinburgh Brain Bank, Academic Department of Neuropathology, University of Edinburgh, Edinburgh, UK; f5 Edinburgh Pathology, University of Edinburgh, Edinburgh, UK; f6 Anne Rowling Regenerative Neurology Clinic, University of Edinburgh, Edinburgh, UK; f7 Centre for Brain Development and Repair, inStem, Bangalore, India; f8 Nuffield Department of Clinical Neurosciences, University of Oxford, Oxford, UK

**Keywords:** CSF, ALS, cytotoxicity, *in vitro*, systematic review

## Abstract

Various studies have suggested that a neurotoxic cerebrospinal fluid profile could be implicated in amyotrophic lateral sclerosis. Here, we systematically review the evidence for cerebrospinal fluid cytotoxicity in amyotrophic lateral sclerosis and explore its clinical correlates. We searched the following databases with no restrictions on publication date: PubMed, Embase and Web of Science. All studies that investigated cytotoxicity *in vitro* following exposure to cerebrospinal fluid from amyotrophic lateral sclerosis patients were considered for inclusion. Meta-analysis could not be performed, and findings were instead narratively summarized. Twenty-eight studies were included in our analysis. Both participant characteristics and study conditions including cerebrospinal fluid concentration, exposure time and culture model varied considerably across studies. Of 22 studies assessing cell viability relative to controls, 19 studies reported a significant decrease following exposure to cerebrospinal fluid from patients with amyotrophic lateral sclerosis, while three early studies failed to observe any difference. Seven of eight studies evaluating apoptosis observed significant increases in the levels of apoptotic markers following exposure to cerebrospinal fluid from patients with amyotrophic lateral sclerosis, with the remaining study reporting a qualitative difference. Although five studies investigated the possible relationship between cerebrospinal fluid cytotoxicity and patient characteristics, such as age, gender and disease duration, none demonstrated an association with any of the factors. In conclusion, our analysis suggests that cerebrospinal fluid cytotoxicity is a feature of sporadic and possibly also of familial forms of amyotrophic lateral sclerosis. Further research is, however, required to better characterize its underlying mechanisms and to establish its possible contribution to amyotrophic lateral sclerosis pathophysiology.

## Teaser

Systematic review of *in vitro* evidence suggests that cerebrospinal fluid cytotoxicity is a feature of sporadic and possibly also of familial forms of amyotrophic lateral sclerosis. Further research is, however, required to elucidate the mechanisms underlying its toxicity, and to establish its possible contribution in overall pathophysiology.

## Graphical Abstract

**Graphical Abstract:**
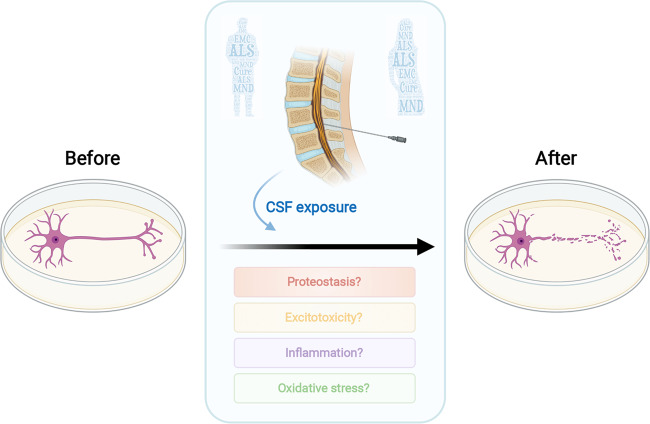

## Introduction

Amyotrophic lateral sclerosis (ALS) is a relentlessly progressive, fatal neurodegenerative condition, characterized by the loss of motor neurons in both the brain and spinal cord, leading to paralysis. Sporadic ALS accounts for the majority of cases (about 90%), while the remaining occurrences, in which the disease is inherited, are known as familial ALS (Brown and Al-Chalabi, 2017). ALS is associated with several genes, for example, *C9ORF72*, *TARDBP*, *SOD1* and *FUS*, with some genes also contributing to the presence of frontotemporal dementia (FTD).

Advances made in the last decade have led to a much improved understanding of ALS pathophysiology, with various mechanisms apparently involved (Hardiman *et al.*, 2017). These include glutamate excitotoxicity, abnormal RNA metabolism, oxidative stress and mitochondrial dysfunction, amongst others. Given the involvement of impaired protein homeostasis, ALS, similar to other neurodegenerative diseases such as Alzheimer’s disease, Parkinson’s disease and Huntington’s disease, is viewed as a proteinopathy, with most cases characterized pathologically by the presence of TAR DNA-binding protein 43 (TDP43)-containing ubiquitinated inclusions (Neumann *et al.*, 2006). One longstanding question, however, pertains to the extent to which each of the above processes contributes to the overall pathophysiology.

Amongst the many lines of enquiry aiming to address this question, several studies have suggested that a neurotoxic cerebrospinal fluid (CSF) profile could be implicated in the disease process (Shaw, 2002; Matias-Guiu *et al.*, 2010; Sumitha *et al.*, 2019; Tokuda *et al.*, 2019). CSF from ALS patients has in fact been shown to exert cytotoxicity *in vitro* (Tikka *et al.*, 2002; Vijayalakshmi *et al.*, 2009; Barber *et al.*, 2011; Sumitha *et al.*, 2019), and to provoke wide-ranging pathology, from neurofilament phosphorylation to musculoskeletal changes, when administered *in vivo* (Shahani *et al.*, 2004; Gomez-Pinedo *et al.*, 2018; Shanmukha *et al.*, 2018). While the cause of these findings remains unclear, they nevertheless suggest the presence of one or more potentially toxic factors in ALS–CSF, with possible involvement in disease spread (Smith *et al.*, 2015). Consistent with this possibility, recent *in vivo* evidence also includes pathological changes being observed distant to the CSF infusion site (Gomez-Pinedo *et al.*, 2018).

A growing body of literature, including various proteomic studies, has helped to demonstrate that CSF composition in ALS may be abnormal (Barschke *et al.*, 2017; Blasco *et al.*, 2017; Hayashi *et al.*, 2019). This includes findings of raised TDP-43 and neurofilament levels, as well as an altered inflammatory profile (Majumder *et al.*, 2018; Schreiber *et al.*, 2018; Gille *et al.*, 2019). Thus, establishing the neurotoxicity of ALS–CSF and its possible determinants could open potential avenues for elucidating the pathophysiology of ALS. We therefore performed a systematic review of *in vitro* studies to review the evidence for CSF cytotoxicity in ALS and also to explore the possible association between cytotoxicity and clinical factors, such as patient age and disease duration.

## Materials and methods

This study has been performed according to the Preferred Reporting Items for Systematic Reviews and Meta-Analyses (PRISMA) guidelines (Moher *et al.*, 2009).

### Search strategy

The following databases were searched on 17 April 2020 to identify relevant studies: PubMed, Embase and Web of Science. No restrictions on publication date were applied. Search terms were as follows:

#### PubMed

(‘amyotrophic lateral sclerosis’ OR ‘ALS’ OR ‘motor neuron’ OR ‘motor neurone’ OR ‘MND’) AND (‘cerebrospinal fluid’ OR ‘CSF’)

#### Embase

(‘amyotrophic lateral sclerosis’ OR ‘ALS’ OR ‘motor neuron’ OR ‘motor neurone’ OR ‘MND’) AND (‘cerebrospinal fluid’ OR ‘CSF’)

#### Web of Science

(‘amyotrophic lateral sclerosis’ OR ‘ALS’ OR ‘motor neuron’ OR ‘motor neurone’ OR ‘MND’) AND (‘cerebrospinal fluid’ OR ‘CSF’)

We also reviewed the reference lists of all eligible studies and screened relevant reviews for potential citations. We collated all the references obtained from these searches and imported them into Endnote X9 for de-duplication.

### Study selection

All studies that investigated cytotoxicity *in vitro* following exposure to CSF from ALS/MND patients were considered for inclusion. Any assay measuring cytotoxicity, cell viability, apoptosis or cell proliferation/cell cycle arrest was accepted. No restrictions on cell line were applied. Studies only investigating morphological and electrophysiological changes or changes in protein expression levels were excluded. *In vivo* and clinical studies were also excluded.

Only studies published in a peer-reviewed journal for which full-text articles are available in the English language were eligible. Reviews, case reports and conference abstracts were not considered for inclusion. Letters to the editor were, however, deemed eligible if sufficient information was provided. One author (KCNKK) screened the title and abstract of each paper. For studies meeting the inclusion criteria, full-text articles were retrieved and two authors (KCNKK and ARM) independently checked the studies for eligibility.

### Data extraction

The primary outcome for this study was *in vitro* cytotoxicity, while secondary outcomes included the clinical correlates of CSF cytotoxicity, namely, the relationship between CSF cytotoxicity and patient characteristics including age, gender, disease duration, disease severity, survival time and site of onset. Data on these were independently extracted from studies by two authors (KCNKK and ARM). One author (KCNKK) extracted data pertaining to study characteristics, which included the following: author, year of publication, country, participant characteristics of both ALS subjects and control subjects (including sample size, type of disease, age, gender, disease duration, survival time and site of onset), culture model, CSF concentration (v/v%), CSF diluent and presence of serum, exposure time, study groups, outcomes assessed, assays for outcome assessment and results.

### Quality assessment

Given that no established guidelines currently exist for assessing the quality of *in vitro* studies, we generated a checklist based on modified Collaborative Approach to Meta-Analysis and Review of Animal Data from Experimental Studies (CAMARADES) criteria (Macleod *et al.*, 2004; Mehta *et al.*, 2019), with additional criteria drawn from the Biospecimen Reporting for Improved Study Quality (BRISQ) tool (Moore *et al.*, 2011). Each study was assigned an overall quality score (total possible score = 9), with one point being given for each of the following: peer-reviewed publication, appropriate study approval stated, appropriate control group identified, procurement and maintenance of *in vitro* model described, appropriate description of CSF preservation process, storage temperature of CSF provided, blinded assessment of outcome stated, number of replicates performed stated and statement of potential conflict of interests.

### Data synthesis

Appropriate summary statistics, including mean difference, standardized mean difference, odds ratio and risk ratio could not be calculated from the extracted data, due to outcome measures (such as cell count) not commonly being reported by included studies, as well as heterogeneity being observed with respect to the interpretation of results. Thus, although a meta-analysis was initially planned as part of our analysis, we decided to summarize our results narratively. We first tabulated and described study characteristics for all studies, before summarizing effectiveness results for the different outcomes including cytotoxicity/cell viability and apoptosis. *P*-values were calculated where it was possible to confidently do so. The clinical correlates of CSF cytotoxicity were also described narratively.

### Data availability statement

All data have been published in this manuscript.

## Results

**Figure 1:**
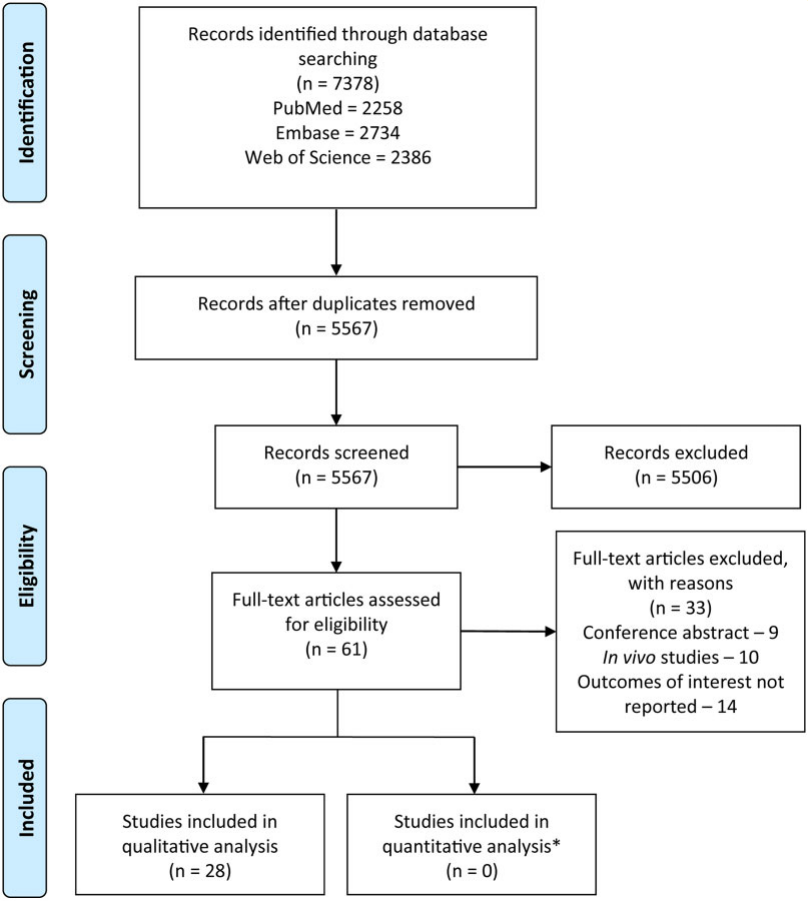
**Preferred reporting items for systematic reviews and meta-analyses (PRISMA) flow diagram.** Chart describes each stage of the study selection process. *Quantitative analysis could not be performed due to outcome measures required to calculate appropriate summary statistics not commonly being reported by included studies. Results were also interpreted differently across studies, and thus could not be pooled together.

We identified a total of 7378 records through database searching which, after de-duplication, reduced to 5567 results (Fig. 1). After screening of title and abstract, 61 articles remained, of which 33 were conference abstracts, *in vivo* studies or did not report our outcomes of interest, and were therefore excluded. Thus, our analysis included 28 studies, conducted across various countries, such as USA, France, Japan, India and Spain (Table 1 shows the study characteristics of included studies).

**Table 1:** Study characteristics of included studies

Author/s (year of publication)	Country	ALS patient population	Control subjects	Study groups	CSF concentration (v/v%)	**CSF diluent** ^a^	Exposure time	Culture model	Quality score
Askanas *et al.* (1981)	USA	10 patients with ALS	7 neuromuscular control patients (NC) 2 non-neuromuscular control patients (NNC)	- Cells exposed to ALS–CSF - Cells exposed to NC–CSF - Cells exposed to NNC–CSF - Untreated cells	50, 75	Culture medium	8 days	Rat spinal cord culture	4
Silani *et al.* (1987)	Italy	3 patients with sporadic ALS	4 control patients (Con)	- Cells exposed to ALS–CSF - Cells exposed to Con–CSF - Untreated cells	10	DMEM (+)	24 h	Human fetal motor cortex culture	5
Crawford *et al.* (1988)	USA	4 patients with ALS	None	- Cells exposed to ALS–CSF - Untreated cells	1.25, 2.5, 5, 10, 37.5	Culture medium	21 days	Rat spinal motor neuron culture	4
Couratier *et al.* (1993)	France	10 patients with ALS	10 neurodegenerative control patients (NC) 10 non-neurodegenerative control patients (NNC)	- Cells exposed to ALS–CSF - Cells exposed to NC–CSF - Cells exposed to NNC–CSF - Untreated cells	10, 20, 50	MEM Earle’s salts (+)	24 h	Rat cortical neuron culture	5
Couratier *et al.* (1994)	France	8 patients with ALS	8 non-neurodegenerative control patients (NNC)	- Cells exposed to ALS–CSF - Cells exposed to NNC–CSF - Untreated cells	50	MEM Earle’s salts (+)	24 h	Rat cortical neuron culture	5
Iwasaki *et al.* (1995)	Japan	10 patients with ALS	10 neurodegenerative control patients (NC) 10 non-neurodegenerative control patients (NNC)	- Cells exposed to ALS–CSF - Cells exposed to NC–CSF - Cells exposed to NNC–CSF	10, 25, 50	DMEM (+)	8 days	Rat spinal cord culture	5
Terro *et al.* (1996)	France	7 patients with ALS	7 non-neurodegenerative control patients (NNC)	- Cells exposed to ALS–CSF - Cells exposed to NNC–CSF - Untreated cells	20	HBSS (+)	48 h	Rat cortical neuron culture	5
Smith *et al.* (1998)	USA	13 sporadic ALS patients with high CSF HNE levels 14 sporadic ALS patients with low CSF HNE levels	None	- Cells exposed to high HNE ALS–CSF - Cells exposed to low HNE ALS–CSF	1, 10	Culture medium	48 h	VSC 4.1 cell line	7
Tikka *et al.* (2002)	Finland	5 ALS patients homozygous for the D90A CuZn-SOD mutation 5 patients with familial ALS 16 patients with sporadic ALS	24 neurological control patients (NC)	- Cells exposed to D90A ALS–CSF - Cells exposed to fALS–CSF - Cells exposed to sALS–CSF - Cells exposed to NC–CSF - Untreated cells	25	DMEM (+)	24 h	Rat spinal cord culture	8
Sen *et al.* (2005)	India	10 patients with ALS	10 neurological control patients (NC)	- Cells exposed to ALS–CSF - Cells exposed to NC–CSF - Untreated cells	10	Eagle’s MEM (+)	24 h	Rat spinal cord culture	7
Anneser *et al.* (2006)	Germany	12 patients with sporadic ALS	6 control patients (Con)	- Cells exposed to ALS–CSF - Cells exposed to Con–CSF - Untreated cells	10	L-15 medium (+)	24 h	Chick motor neuron culture and chick mixed spinal cord culture	5
Shobha *et al.* (2007)	India	5 patients with ALS	5 neurological control patients (NC)	- Cells exposed to ALS–CSF - Cells exposed to NC–CSF - Untreated cells	10	DMEM (−)	48 h	Rat mixed spinal cord culture	7
Vijayalakshmi *et al.* (2009)	India	5 patients with sporadic ALS	5 neurological control patients (NC)	- Cells exposed to ALS–CSF - Cells exposed to NC–CSF - Untreated cells	10	DMEM (+)	48 h	NSC-34 cell line	7
Fiszman *et al.* (2010)	Argentina	6 patients with sporadic ALS	3 control patients (Con)	- Cells exposed to ALS–CSF - Cells exposed to Con–CSF - Untreated cells	50	Locke’s solution (−)	24 h	Mouse cortical neuron culture	7
Barber *et al.* (2011)	UK	10 patients with ALS	10 control patients (Con)	- Cells exposed to ALS–CSF - Cells exposed to Con–CSF - Untreated cells	20, 50	Neurobasal medium (−)	24 h	Rat motor neuron culture	8
Kulshreshtha *et al.* (2011)	India	6 patients with ALS	6 neurological control patients (NC)	- Cells exposed to ALS–CSF - Cells exposed to NC–CSF - Untreated cells	10	DMEM (+)	48 h	NSC-34 cell line	7
Vijayalakshmi *et al.* (2011)	India	5 patients with sporadic ALS	5 neurological control patients (NC)	- Cells exposed to ALS–CSF - Cells exposed to NC–CSF - Untreated cells	10	DMEM (+)	48 h	NSC-34 cell line	6
Yáñez *et al.* (2011)	Spain	27 patients with ALS	14 control patients (Con)	- Cells exposed to ALS–CSF - Cells exposed to Con–CSF - Untreated cells	10	Neurobasal medium (−)	24 h	Rat cortical neuron culture	8
Varghese *et al.* (2013)	India	16 patients with ALS	13 control patients (Con)	- Cells exposed to ALS–CSF - Cells exposed to Con–CSF - Untreated cells	10	DMEM (+)	48 h	NSC-34 cell line	8
Gomez-Pinedo *et al.* (2014)	Spain	3 patients with ALS	3 control patients (Con)	- Cells exposed to ALS–CSF - Cells exposed to Con–CSF - Untreated cells	10	Neurobasal medium (−)	24 h	Rat cortical neuron culture	7
Yáñez *et al.* (2014)	Spain	17 patients with ALS	None	- Cells exposed to ALS–CSF - Untreated cells	10	Neurobasal medium (−)	24 h	Rat cortical neuron culture	4
Ding *et al.* (2015)	China	18 patients with sporadic ALS 8 patients with sporadic ALS and FTD	15 non-neurological control patients (NNC)	- Cells exposed to ALS–CSF - Cells exposed to ALS–FTD–CSF - Cells exposed to NNC–CSF	30	DMEM (+)	21 days	U251 cell line	8
Sharma *et al.* (2015)	India	10 patients with ALS	10 control patients (Con)	- Cells exposed to ALS–CSF - Cells exposed to Con–CSF - Untreated cells	10	DMEM (+)	48 h	NSC-34 cell line	8
Vijayalakshmi *et al.* (2015)	India	5 patients with sporadic ALS	5 neurological control patients (NC)	- Cells exposed to ALS–CSF - Cells exposed to NC–CSF - Untreated cells	10	DMEM (+)	48 h	NSC-34 cell line	7
Galán *et al.* (2017)	Spain	31 patients with ALS	None	- Cells exposed to ALS–CSF - Untreated cells	10	Neurobasal medium (−)	24 h	Rat cortical neuron culture	7
Shruthi *et al.* (2017)	India	5 patients with ALS	5 neurological control patients (NC)	- Cells exposed to ALS–CSF - Cells exposed to NC–CSF - Untreated cells	10	DMEM (+)	48 h	NSC-34 cell line	7
Sumitha *et al.* (2019)	India	5 patients with sporadic ALS	5 neurological control patients (NC)	- Cells exposed to ALS–CSF - Cells exposed to NC–CSF - Untreated cells	10	DMEM (−)	48 h	hESC-derived motor neuron culture	7
Tokuda *et al.* (2019)	Japan	10 patients with sporadic ALS 1 patient with familial SOD1–ALS	15 neurodegenerative control patients (NC) 11 non-neurodegenerative control patients (NNC)	- Cells exposed to ALS–CSF - Cells exposed to NC–CSF - Cells exposed to NNC–CSF - Untreated cells	10	DMEM (+)	48 h	NSC-34 cell line	8

a Serum presence and serum-free conditions are indicated in brackets as ‘+’ and ‘−’, respectively.

DMEM: Dulbecco's Modified Eagle Medium; fALS: familial amyotrophic lateral sclerosis; FTD: frontotemporal dementia; HBSS: Hanks’ balanced salt solution; hESC: human embryonic stem cell; HNE: 4‐hydroxynonenal; MEM: Minimal Essential Medium; NSC-34: mouse spinal cord-neuroblastoma hybrid cell line; SOD1: superoxide dismutase 1; U251: human glioma cell line; VSC 4.1: cholinergic cAMP-differentiated motor neuron-neuroblastoma hybrid cell line.

### Subject characteristics

The sample size of ALS subjects ranged from 3 to 31, with different ALS patient populations being studied, some of which include sporadic ALS, familial ALS, as well as patients with both ALS and FTD. Different criteria were also applied to ALS patient selection, since some studies included both subjects diagnosed with ‘probable’ and ‘definite’ ALS, while other studies limited participants to those with ‘definite’ ALS (see Supplementary Table 1 for additional information on participant characteristics including: disease description, age, gender, disease duration, survival time and site of onset). Control groups varied across studies, and, where possible, we broadly classified them into categories, such as ‘neurological control’, ‘neurodegenerative control’, ‘non-neurological control’ and ‘non-neurodegenerative control’. Due to ethical implications, control groups generally did not involve healthy individuals. Four studies did not include a control group.

Reporting of subject characteristics, including age, gender, disease duration, survival time and site of onset was inconsistent across studies, with the data less likely to be available for earlier studies. In studies that reported these, subjects were generally aged between around 45 and 75 years old, while gender ratio varied considerably from study to study. The clinical course was described by either disease duration or survival time, although survival times were sometimes inexact in cases where some patients were still alive. A number of studies further reported the site of onset, namely, whether the disease was limb onset or bulbar onset. Other features that varied across studies include whether the subjects were drug-naïve at the time of lumbar puncture and whether age- and gender-matching were possible.

### Study conditions

Heterogeneity was also observed with regard to study conditions. Different CSF concentrations ranging from 1% to 75% were used to assess cytotoxicity, while CSF exposure time also varied from 24 h to 21 days. The most common CSF concentration and exposure times, however, were 10% and 24 or 48 h, respectively. CSF was diluted in culture media such as Dulbecco's Modified Eagle Medium in all but two studies, where the diluents were Hanks’ balanced salt solution (Terro *et al.*, 1996) and Locke’s solution (Fiszman *et al.*, 2010). Serum was present in 17 studies, while eight studies observed serum-free conditions. The presence of serum could not be ascertained in the remaining three studies. *In vitro* culture models included spinal cord cultures, cortical neuron cultures and motor neuron cultures, as well as cell lines, such as NSC-34 (mouse spinal cord-neuroblastoma hybrid cell line), VSC 4.1 (cholinergic cAMP-differentiated motor neuron-neuroblastoma hybrid cell line) and U251 (human glioma cell line). One recent study also assessed cytotoxicity in motor neurons differentiated from human embryonic stem cells (Sumitha *et al.*, 2019).

### Quality assessment

Overall quality score of individual studies ranged from 4 to 8, with lower quality scores generally being observed in earlier studies (Supplementary Table 2). Most studies (24 out of 28) included one or more control groups. Procurement and maintenance of the *in vitro* model, as well as CSF preservation and storage temperature, were also usually described. Blinded outcome assessment was, however, rarely carried out, with only six studies stating that outcomes were blindly assessed.

### Cytotoxicity/cell viability

Twenty-four studies assessed cytotoxicity/cell viability through various techniques, including cell counting, trypan blue exclusion, MTS assay, 3-(4,5-dimethylthiazol-2-yl)-2,5-diphenyltetrazolium bromide (MTT) assay and lactate dehydrogenase (LDH) assay (Table 2). Nineteen studies observed a significant difference in cell viability following exposure to ALS–CSF compared to controls. The significance level across studies varied from *P* < 0.05 to *P* < 0.001, with the *P*-value not being specified in two studies. In one of these studies, however, the difference was only significant at 50% CSF concentration, but not at a concentration of 20% (Barber *et al.*, 2011). Another study also failed to observe a decrease in neuronal survival at a 24-h time point, but only reported the difference as significant on day 10 (Silani *et al.*, 1987). Three early studies did not find any significant difference in cytotoxicity following exposure to ALS–CSF compared to controls (Askanas *et al.*, 1981; Crawford *et al.*, 1988; Iwasaki *et al.*, 1995). Two studies also assessed cytotoxicity within ALS patient populations. In the first study, which evaluated CSF cytotoxicity in 31 patients with ‘probable’ or ‘definite’ ALS at the time of diagnosis, 67.7% of patients were considered to possess cytotoxic CSF, with cytotoxicity being defined as a reduction in cell survival greater or equal to 25% compared to control (Galán *et al.*, 2017). The second study compared CSF cytotoxicity between sporadic ALS patients with a high concentration of 4‐hydroxynonenal with those with a low concentration of 4‐hydroxynonenal, and reported a significant difference at both 1% and 10% CSF concentration (Smith *et al.*, 1998).

**Table 2:** Summary of findings of included studies

Author/s (year of publication)	Outcome/s assessed	Assay/s for assessing outcome	Results
Askanas *et al.* (1981)	Cell viability	NSE radioimmunoassay	Little evidence for toxic effect of CSF suggested by slight decrease (∼9%) in enolase activity of CSF-treated cultures (ALS and disease controls) compared with untreated cultures
Silani *et al.* (1987)	Cell viability	NS	No obvious decrease in neuronal survival following exposure to ALS–CSF at 24 h. Neuronal cell loss only observed at day 5, with the decrease becoming significant at day 10
Crawford *et al.* (1988)	Cell viability	NS	No change in motor neuron survival observed following exposure by ALS–CSF
Couratier *et al.* (1993)	Cell viability	Cell counting	Significant decrease in neuronal survival following exposure to 50% ALS–CSF compared to controls (*P* < 0.001). A smaller decrease in survival was observed at more dilute ALS–CSF concentrations (20% and 10%)
Couratier *et al.* (1994)	Cell viability	Cell counting, FDA staining	Significant decrease in neuronal survival following exposure to ALS–CSF compared to controls (*P* < 0.001)
Iwasaki *et al.* (1995)	Cell viability	Cell counting	No significant differences in neuronal survival following exposure to ALS–CSF compared to controls at any CSF concentration
Terro *et al.* (1996)	Cell viability	Cell counting, FDA and PI double staining	Significant decrease in neuronal survival following exposure to ALS–CSF compared to controls (*P* < 0.001)
Smith *et al.* (1998)	Cell viability	Trypan blue staining, MTS assay	Significant difference in VSC 4.1 cell survival between samples exposed to high HNE ALS–CSF and low HNE ALS–CSF both at 1% CSF and 10% CSF (*P* < 0.001)
Tikka *et al.* (2002)	Cell viability, apoptosis	Cell counting, bis-benzimide staining	Significant increase in the proportion of apoptotic neurons and significant decrease in the percentage of surviving neurons following exposure to D90A ALS–CSF, fALS–CSF and sALS–CSF compared to controls (*P* < 0.05)
Sen *et al.* (2005)	Cell viability	Live/dead cell assay (calcein-AM and ethidium homodimer)	Significant decrease in both motor neuron survival and survival of other spinal neurons following exposure to ALS–CSF compared to controls (*P* < 0.001). Significant difference between motor neuron survival compared to survival of other spinal neurons also observed (*P* < 0.001)
Anneser *et al.* (2006)	Cell viability, apoptosis	Cell counting, trypan blue staining, PI/DAPI staining, TUNEL assay	Significant increase in apoptotic cells and significant decrease in motor neuron survival following exposure to ALS–CSF compared to controls (*P* < 0.001). Significant decrease in survival also observed in mixed spinal cord culture following exposure to ALS–CSF (*P*-value not specified)
Shobha *et al.* (2007)	Cell viability	LDH assay	Increased LDH activity following exposure to ALS–CSF compared to controls (*P*-value not specified)
Vijayalakshmi *et al.* (2009)	Cell viability	MTT assay, LDH assay	Significant reduction in viability of NSC-34 cells (*P* < 0.001) and significant increase in LDH activity (*P* < 0.01) following exposure to ALS–CSF compared to controls
Fiszman *et al.* (2010)	Cell viability	Cell counting, trypan blue staining	Significant decrease in neuronal survival following exposure to ALS–CSF compared to controls (*P* < 0.05)
Barber *et al.* (2011)	Cell viability	Cell counting	Significant decrease in motor neuron survival following exposure to 50% ALS–CSF (*P* < 0.05) and 50% Con–CSF (*P* < 0.005) compared to untreated samples. Decrease in motor neuron survival, however, not significant at 20% ALS–CSF and 20% Con–CSF
Kulshreshtha *et al.* (2011)	Cell viability	LDH assay	Significant increase in LDH activity following exposure to ALS–CSF compared to controls (*P* < 0.01)
Vijayalakshmi *et al.* (2011)	Apoptosis	TUNEL assay	TUNEL-positive nuclei observed in cells exposed to ALS–CSF while cells in control groups showed unstained nuclei
Yáñez *et al.* (2011)	Cell viability	MTT assay, LDH assay	Significant decrease in neuronal viability (*P* < 0.001) and significant increase in LDH activity (*P* < 0.05) following exposure to ALS–CSF compared to controls
Varghese *et al.* (2013)	Cell viability	MTT assay, LDH assay	Significant decrease in cell viability and significant increase in LDH activity following exposure to ALS–CSF compared to controls (*P* < 0.001)
Gomez-Pinedo *et al.* (2014)	Apoptosis	Caspase-3 assay	Significant increase in caspase-3 positive cells following exposure to ALS–CSF compared to controls (*P* < 0.05)
Yáñez *et al.* (2014)	Cell viability	MTT assay	Significant decrease in neuronal viability following exposure to ALS–CSF compared to control (*P* < 0.05)
Ding *et al.* (2015)	Apoptosis	Caspase-3 assay, Bcl-2 assay	Significant increase in cleaved caspase-3 levels following exposure to ALS–CSF (*P* < 0.05) and ALS–FTD–CSF (*P* < 0.01) compared to control. Significant decrease in Bcl-2 levels following exposure to ALS–FTD–CSF compared to both ALS–CSF and control (*P* < 0.05)
Sharma *et al.* (2015)	Cell viability	MTT assay	Significant decrease in neuronal viability following exposure to ALS–CSF compared to controls (*P* < 0.001)
Vijayalakshmi *et al.* (2015)	Apoptosis	TUNEL assay, caspase-3 assay	Significant increase in proportion of TUNEL-positive cells and expression of caspase-3 following exposure to ALS–CSF compared to controls (*P* < 0.001)
Galán *et al.* (2017)	Cell viability	MTT assay	CSF cytotoxicity was observed in 21 patients (67.7%) while the remaining 10 patients (32.3%) were considered to possess non-cytotoxic CSF (cytotoxicity was defined as a decrease in neuronal viability greater or equal to 25% compared to control)
Shruthi *et al.* (2017)	Cell viability, apoptosis	MTT assay, caspase-3 assay	Significant decrease in cell viability (*P* < 0.05) and significant increase in caspase-3 expression (*P* < 0.05) following exposure to ALS–CSF compared to controls
Sumitha *et al.* (2019)	Cell viability, apoptosis	MTT assay, LDH assay, caspase-9 assay, Bcl-2 assay, Bax assay	Significant decrease in neuronal viability and significant increase in LDH activity following exposure to ALS–CSF compared to controls (*P* < 0.001). Non-significant increase in proportion of BCL2-positive neurons but significant increases in proportion of Bax-positive and caspase-9-positive neurons (*P* < 0.05) following exposure to ALS–CSF compared to controls
Tokuda *et al.* (2019)	Cell viability	Cell Counting Kit-8	Significant decrease in cell viability following exposure to ALS–CSF compared to controls (*P* < 0.01)

DAPI: 4′,6-diamidino-2-phenyindole, diacetate; fALS: familial amyotrophic lateral sclerosis; FDA: fluorescein diacetate; FTD: frontotemporal dementia; HNE: 4‐hydroxynonenal; LDH: lactate dehydrogenase; MTT: 3-(4,5-dimethylthiazol-2-yl)-2,5-diphenyltetrazolium bromide; NS: not specified; NSC-34: mouse spinal cord-neuroblastoma hybrid cell line; NSE: neuron-specific enolase; PI: propidium iodide; sALS: sporadic amyotrophic lateral sclerosis; TUNEL: terminal deoxynucleotidyl transferase dUTP nick end labelling; VSC 4.1: cholinergic cAMP-differentiated motor neuron-neuroblastoma hybrid cell line.

### Apoptosis

Apoptosis was investigated by eight studies via techniques such as terminal deoxynucleotidyl transferase dUTP nick end labelling (TUNEL) assay, caspase-3 assay, caspase-9 assay, Bcl-2 assay and Bax assay. Seven of these studies were quantitative in nature and observed significant increases in the levels of different apoptotic markers, including caspase-3 and caspase-9, following exposure to ALS–CSF compared to controls. However, the increase in Bcl-2 levels following ALS–CSF exposure did not reach statistical significance (Ding *et al.*, 2015; Sumitha *et al.*, 2019). The remaining study, which only qualitatively evaluated apoptosis, reported the presence of TUNEL-stained nuclei in cells exposed to ALS–CSF, but not in those from control groups (Vijayalakshmi *et al.*, 2011). We also note that ALS–FTD–CSF produced a significant increase in caspase-3 expression compared to ALS–CSF, although it was found to result in a significant decrease in Bcl-2 levels (Ding *et al.*, 2015).

### Clinical correlates

Five studies further assessed the possible relationship between CSF cytotoxicity and patient characteristics, including age, disease duration, disease severity, survival time and site of onset (Table 3). However, none of the studies demonstrated an obvious association between CSF cytotoxicity and any of the listed factors. Although one study reported differences based on age, gender and site of onset, the difference was not statistically significant (Yáñez *et al.*, 2011). Another study also reported very low apoptotic activity in patients with the longest survival times, but a clear correlation was again not established (Barber *et al.*, 2011).

**Table 3:** Clinical correlates of ALS–CSF cytotoxicity

Author/s (year of publication)	Patient characteristic	Results
Tikka *et al.* (2002)	Survival time	Little correlation observed between CSF cytotoxicity and patient survival time. Considerably lower apoptotic activity was, however, observed in the two patients with the longest survival times
Anneser *et al.* (2006)	Age, disease duration	Little correlation observed between CSF cytotoxicity and age (*r* = −0.22) or disease duration prior to lumbar puncture (*r* = −0.24)
Barber *et al.* (2011)	Age, gender, disease duration	Little correlation observed between CSF cytotoxicity and age or disease duration. CSF cytotoxicity was also not influenced by gender
Yáñez *et al.* (2011)	Age, gender, disease duration, disease severity, site of onset	Although female patients, younger patients and patients with bulbar onset ALS appeared to possess more cytotoxic CSF, the differences did not reach statistical significance. No relationship was also observed between CSF cytotoxicity and disease duration or disease severity
Galán *et al.* (2017)	Age, gender, disease duration, survival time, site of onset	No significant differences were observed between patients with cytotoxic CSF and those with non-cytotoxic CSF with respect to age, gender, disease duration, survival time or site of onset

## Discussion

Our summary of findings in this study suggests that CSF cytotoxicity is a feature of sporadic and also possibly of familial ALS, with ALS–CSF appearing to exert cytotoxicity at a wide range of concentrations and exposure times, as well as across different culture models. Although a few studies failed to observe cytotoxicity following exposure to ALS–CSF compared to controls, we note that these were generally earlier studies (Askanas *et al.*, 1981; Silani *et al.*, 1987; Crawford *et al.*, 1988; Iwasaki *et al.*, 1995). Our results also indicate an apoptotic component to ALS–CSF, with greater apoptosis being observed in cells following exposure to CSF from patients with both ALS and FTD (Ding *et al.*, 2015). However, we fail to demonstrate a connection between CSF cytotoxicity and patient characteristics, such as age, gender, disease duration and survival time. The cause of this lack of association remains unclear, but suggests that CSF cytotoxicity may not be appropriate as a biomarker in ALS.

While the findings from this study support the cytotoxicity of ALS–CSF, little consensus exists as to the factors underlying it. Different potential candidates have been suggested to explain ALS–CSF cytotoxicity, with glutamate being an important example, given the potentially raised glutamate levels in ALS patients (Spreux-Varoquaux *et al.*, 2002; Fiszman *et al.*, 2010). However, although glutamate antagonists have been shown to have a protective effect on CSF neurotoxicity, less evidence is available to support the toxicity of glutamate endogenous to ALS–CSF (Couratier *et al.*, 1994; Tikka *et al.*, 2002; Anneser *et al.*, 2006; Matias-Guiu *et al.*, 2010). Riluzole, despite its modest clinical benefit, also failed to confer neuroprotection *in vitro* (Yáñez *et al.*, 2011).

Another potential mechanism underlying CSF-induced neurodegeneration, given the increasing recognition of ALS as a proteinopathy and the growing evidence supporting the prion-like properties of several key ALS proteins, notably that of TDP-43 and SOD1 (Brauer *et al.*, 2018), is proteostasis. This is supported by findings demonstrating that CSF containing misfolded SOD1 could trigger neurodegeneration in NSC-34 cells (Tokuda *et al.*, 2019). Another recent study further revealed that ALS–CSF could promote TDP-43 proteinopathy both following *in vitro* exposure and *in vivo* injection, although the observed changes were much more pronounced in TDP-43 transgenic mice, than in normal mice (Mishra *et al.*, 2020). Other ALS proteins that have been suggested to possess prion-like properties include FUS and C9ORF72-associated dipeptide repeat proteins (Nomura *et al.*, 2014; Westergard *et al.*, 2016), but whether they could exert toxicity at levels comparable to that of ALS–CSF is unclear.

Consistent with the non-cell autonomous component of ALS pathophysiology (Zhao *et al.*, 2020), ALS–CSF toxicity was also found to extend to glial cells. Notably, exposure to ALS–CSF has been found to result in pro-inflammatory activity, both in astrocytes and microglia (Mishra *et al.*, 2016, 2017). Furthermore, ALS–CSF was also found to produce different changes in motor neurons co-cultured with glia than in motor neuron mono-cultures (Barber *et al.*, 2011), although the reasons for this disparity remain to be established. Highlighting the potentially inflammatory aspect of ALS–CSF, a number of immune components and growth factors have also been linked to CSF cytotoxicity (Matias-Guiu *et al.*, 2010).

Acknowledging that the pathophysiology of ALS remains unknown, characterizing cytotoxicity associated with ALS–CSF could greatly improve our understanding of the mechanisms responsible for neurodegeneration in ALS patients. These insights could arise from additional studies employing a proteomic approach, given that few such studies have been performed so far (Varghese *et al.*, 2013). *In vitro* results also need to be complemented by *in vivo* evidence, which have to date revealed various changes following CSF infusion, including neurofilament phosphorylation, endoplasmic reticulum stress, as well as motor dysfunction (Deepa *et al.*, 2011; Vijayalakshmi *et al.*, 2011; Shanmukha *et al.*, 2018). Although the observed changes have been reported to be histologically similar to sporadic ALS cases (Gomez-Pinedo *et al.*, 2018), whether CSF toxicity studies accurately capture the mechanisms involved in ALS pathophysiology, and could serve as an important model for ALS, is not yet clear. Various lines of evidence, however, hint at a potential contribution of CSF in the spread of the disease in ALS patients, with one major appeal for this model being its explanatory potential with respect to clinical observations (Smith *et al.*, 2015).

Indeed, despite numerous models having been posited to explain disease evolution in the context of ALS, clinical observations remain incompletely explained. In addition to being highly heterogeneous, the disease is occasionally found to spread in a non-contiguous manner, with the onset believed to be multifocal in nature (Sekiguchi *et al.*, 2014). Trans-synaptic spread and cell-to-cell transmission via exosomes have both been suggested as possible mechanisms of spread (Braak *et al.*, 2013; Iguchi *et al.*, 2016). With the evidence for exosome transmission still a topic of debate (Brauer *et al.*, 2018), future research aimed at uncovering its possible contribution could play an important role in determining the importance of CSF circulation as a route of spread. Necroptosis, in which the contents of the dying cell are released into the surrounding environment, is another possible mechanism that may deserve investigation (Ito *et al.*, 2016).

Intriguingly, while considerably more literature surrounds CSF cytotoxicity in the context of ALS, this does not appear to be a feature specific to ALS. Similar to ALS, the neurotoxicity of CSF from patients with Parkinson’s disease patients has been demonstrated as early as 1999, with degeneration of dopaminergic neurons being observed *in vitro* (Le *et al.*, 1999). This finding has also been confirmed more recently by a different group (Kong *et al.*, 2015). Furthermore, we also found that, in some of the studies included in this review, CSF from control groups could also exert cytotoxicity. For instance, one study, in which ALS–CSF cytotoxicity was assessed alongside CSF from 10 control subjects undergoing lumbar puncture to exclude subarachnoid haemorrhage or viral meningitis, demonstrated significantly increased cytotoxicity following exposure to CSF from control subjects compared with ALS–CSF (Barber *et al.*, 2011).

Nevertheless, although we consider our findings to be suggestive of the cytotoxicity of ALS–CSF, we acknowledge a number of limitations in this review. First, included studies assessed cytotoxicity in different cell lines, some of which, including the VSC 4.1 and U251 cell lines, could potentially have responded differently to CSF exposure compared to motor neurons. The NSC-34 cell line, which has been used by many studies to study CSF cytotoxicity has also been subject to controversy in the past (Hornburg *et al.*, 2014). Given the qualitative nature of this study, it was not possible to investigate the difference in vulnerability between cortical and spinal neurons to CSF cytotoxicity. The variety of cell lines employed, some of which could differ considerably from human motor neurons in their response to CSF exposure, is also a major limitation of our study. Notwithstanding this, one recent study that post-dates our literature search, in which human iPSC-derived spinal motor neurons were exposed to ALS–CSF, supports the cytotoxicity of ALS–CSF towards motor neurons (Brauer *et al.*, 2020).

Second, the considerable heterogeneity with regard to reporting of study results and their interpretation meant that we were unable to conduct a meta-analysis. Reporting of study characteristics and participant details was also variable, and we therefore failed to further analyse the association between patient characteristics and CSF cytotoxicity. Notably, other features of ALS, including *C9ORF72* status and the presence of cognitive or behaviour change were not commonly assessed in study populations, and could therefore be considered in future studies. The time point at which lumbar puncture was performed, namely, whether it was part of diagnostic work-up earlier in disease trajectory, or later in disease course, was also not always stated. Finally, the contribution of study conditions, including CSF concentration and exposure time, could also be more extensively investigated. Although the CSF diluent and the presence of serum did not appear to influence CSF cytotoxicity overall, a definite conclusion cannot be drawn.

Moving forwards, we believe that there is a need for future studies assessing CSF cytotoxicity to provide an accurate record of study procedures and ensure greater consistency in reporting of study results, in order to facilitate meta-analysis of research findings. We thus present a checklist (Supplementary Table 3), which, though not intended as a comprehensive guideline, includes items of possible relevance to the assessment of CSF cytotoxicity. Disease staging, for instance, despite its known association with CSF composition, is not always reported by studies. In particular, we recommend that, with respect to study results, steps involved in the calculation of the summary statistic, as well as the study groups involved, are clearly described, with the raw data made available for possible future analyses.

## Conclusion

The lack of success in finding a possible treatment for ALS, with the only globally licenced drug for ALS so far being riluzole (Bensimon *et al.*, 1994), has led to various lines of enquiry aimed at identifying the processes underlying ALS pathophysiology. Here, we performed a qualitative assessment of the existing literature, the outcome of which suggests that CSF cytotoxicity, a feature which has previously been linked to ALS pathophysiology, can be observed in sporadic and possibly also in familial forms of ALS. Thus, improving our understanding of the mechanisms responsible for CSF cytotoxicity, and, importantly, establishing their possible contribution in ALS pathophysiology, could play a potential role in future ALS research.

## Supplementary Material

fcaa121_Supplementary_Data

## Abbreviations

ALS =: amyotrophic lateral sclerosis
BRISQ =: Biospecimen Reporting for Improved Study Quality
C9ORF72 =: chromosome 9 open reading frame 72
CAMARADES =: Collaborative Approach to Meta-Analysis and Review of Animal Data from Experimental Studies
CSF =: cerebrospinal fluid
DAPI =: 4′,6-diamidino-2-phenyindole
fALS =: familial amyotrophic lateral sclerosis
FDA =: fluorescein diacetate
FTD =: frontotemporal dementia
FUS =: fused-in-sarcoma
hESC =: human embryonic stem cell
HNE =: 4-hydroxynonenal
LDH =: lactate dehydrogenase
MND =: motor neuron disease
MTT =: 3-(4,5-dimethylthiazol-2-yl)-2,5-diphenyltetrazolium bromide
NSC-34 =: mouse spinal cord-neuroblastoma hybrid cell line
NSE =: neuron-specific enolase
PI =: propidium iodide
PRISMA =: Preferred Reporting Items for Systematic Reviews and Meta-Analyses
sALS =: sporadic amyotrophic lateral sclerosis
SOD1 =: superoxide dismutase 1
TDP-43 =: TAR DNA binding protein 43
TUNEL =: terminal deoxynucleotidyl transferase dUTP nick end labelling
U251 =: human glioma cell line
VSC 4.1 =: cholinergic cAMP-differentiated motor neuron-neuroblastoma hybrid cell line
