# Supplementary material for: Cerebrospinal fluid cytotoxicity in amyotrophic lateral sclerosis: a systematic review of in vitro studies
Source: Brain Commun. 2020 Aug 6;2(2):fcaa121. doi: 10.1093/braincomms/fcaa121 (PMC7566327; doi:10.1093/braincomms/fcaa121)
Supplement: fcaa121_Supplementary_Data [file fcaa121_supplementary_data.pdf]

Supplementary Table S1. Participant characteristics

Author/s (Year of publication)	Patient population	Control subjects	Age	Gender ratio (% female)	Disease duration/ Survival time (months)	Site of onset
**Askanas *et al.* (1981)**	10 patients with classical ALS	7 neuromuscular control patients (3 with neuropathy, 1 with progressive spinal atrophy, 1 with myotonic atrophy<br>1 patient with ophthalmoplegia and seizures and 1 with morphologically nonspecific myopathy)<br>2 non-neuromuscular control patients	NA	NA	NA	NA
**Silani *et al.* (1987)**	3 patients with sporadic ALS	4 control patients	NA	NA	NA	NA
**Swift *et al.* (1988)**	4 patients with classical ALS	None	NA	NA	NA	NA
**Couratier *et al.* (1993)**	10 patients with classical ALS	10 age-matched neurodegenerative control patients (4 with Parkinson's disease, 2 with supranuclear palsy, 2 with normal pressure hydrocephalus, 1 with Friedreich's ataxia and 1 with senile dementia) (NC)<br>10 age-matched non-neurodegenerative control patients with lumbar radiculopathy (NNC)	ALS: Range = 45-60<br>NC: Range = 50-82<br>NNC: Range= 45-55	NA	NA	NA
**Couratier *et al.* (1994)**	8 patients with ALS	8 non-neurodegenerative control patients with lumbar radiculopathy	NA	NA	NA	NA
**Iwasaki *et al.* (1995)**	10 patients with classical ALS	10 age- and sex-matched neurodegenerative control patients (4 with olivopontocerebellar atrophy, 3 with polymyositis, 2 with myotonic dystrophy and 1 with senile dementia)<br>10 age- and sex-matched non-neurodegenerative control patients with tension headache	ALS: Range = 31-69	NA	NA	NA
**Terro *et al.* (1996)**	7 patients with classical ALS	7 non-neurodegenerative control patients with lumbar radiculopathy (NNC)	ALS: Range = 62-77<br>NNC: Range = 33-54	NA	NA	NA
**Smith *et al.* (1998)**	13 sporadic ALS patients with high CSF HNE levels (Mean $\pm$ SD = 5.35 $\pm$ 3.22 ng/mL)<br>14 sporadic ALS patients with low CSF HNE levels (Mean $\pm$ SD = 0.10 $\pm$ 0.10 ng/mL)	None	NA	NA	NA	NA
**Tikka *et al.* (2002)**	5 ALS patients homozygous for the D90A CuZn-SOD mutation<br>5 patients with familial ALS<br>16 patients with sporadic ALS (all without CuZn-SOD mutations)<br>All ALS patients had 'probable' or 'definite' ALS	24 neurological control patients with diseases such as headache, multiple sclerosis, brain infarction and hydrocephalus (NC)	ALS: Mean $\pm$ SD = 60.0 $\pm$ 8.7<br>NC: Mean $\pm$ SD = 50.0 $\pm$ 16.7	ALS: 38.4%<br>NC: 62.5%	ALS: Mean $\pm$ SD = 57.3 $\pm$ 54.0 (Survival time)	NA
**Sen *et al.* (2005)**	10 patients with ALS	10 neurological control patients	NA	NA	NA	NA
**Anneser *et al.* (2006)**	12 patients with sporadic ALS ('probable' or 'definite')	6 control subjects including patients undergoing spinal tap for exclusion of cerebral haemorrhage and patients with noninflammatory polyneuropathy	ALS: Mean $\pm$ SD = 68.0 $\pm$ 7.7	ALS: 58.3%	ALS: Mean $\pm$ SD = 11.0 $\pm$ 8.9 (Disease duration)	NA
**Shobha *et al.* (2007)**	5 patients with ALS	5 age- and gender-matched non-neurodegenerative control patients with neurological diseases including diabetic cranial neuropathy with recurrent bilateral III nerve palsy, benign intracranial hypertension, ataxic neuropathy, demyelinating spinal cord disorder, cognitive changes in diabetic patient (NC)	ALS: Range = 48-63<br>NC: Range = 48-63	NA	ALS: Mean = 12<br>NC: Mean = 24<br>(Disease duration)	NA

**Vijayalakshmi *et al.* (2009)**	5 patients with sporadic ALS	5 age- and gender-matched non-neurodegenerative control patients with neurological diseases including transverse myelitis, mononeuritis multiplex and ataxic neuropathy	NA	NA	NA	NA
**Fiszman *et al.* (2010)**	6 patients with sporadic ALS ('possible' or 'definite')	3 control patients (Con)	ALS: Mean + SD = 50.2 + 11.0<br>Con: Mean + SD = 47.3 + 21.6	ALS: 33.3%<br>Con: 100%	NA	NA
**Barber *et al.* (2011)**	10 patients with ALS	10 control subjects including patients attending hospital with unexplained headache undergoing lumbar puncture to exclude subarachnoid haemorrhage or viral meningitis (Con)	ALS: Mean + SD = 60.3 + 12.3<br>Con: Mean + SD = 41.8 + 14.2	ALS: 50% Con: 50%	ALS: Mean + SD = 49.7 + 32.9 (Survival time)	7 limb onset<br>3 bulbar onset
**Kulshreshtha *et al.* (2011)**	6 drug-naïve patients with 'definite' ALS	6 age- and gender-matched non-neurodegenerative control patients (2 with metabolic encephalopathy, 2 with idiopathic intracranial hypertension, 1 with systemic infection and encephalopathy and 1 with an axonal variant of Guillain-Barré syndrome)	ALS: Range = 42-55	ALS: 33.3%	ALS: Range = 7-18 (Disease duration)	2 limb onset<br>4 bulbar onset
**Vijayalakshmi *et al.* (2011)**	5 patients with sporadic ALS	5 age- and gender-matched non-neurodegenerative control patients with neurological diseases including transverse myelitis, mononeuritis multiplex and ataxic neuropathy	NA	NA	NA	NA
**Yanez *et al.* (2011)**	27 patients with ALS	14 control patients with undefined headache requiring lumbar puncture for diagnostic purposes (Con)	ALS: Mean (Range) = 59.2 (40-75)<br>Con: Mean (Range) = 43.1 (27-67)	ALS: 51.9%<br>Con: 78.6%	NA	NA
**Varghese *et al.* (2013)**	16 patients with ALS	13 age-matched control patients undergoing spinal anaesthesia for orthopaedic surgery (Con)	ALS: Mean + SD (Range) = 47.38 ± 5.38 (38-54)<br>Con: Mean + SD (Range) = 45.7 ± 7.04 (39-60)	ALS: 37.5% Con: 15.4%	ALS: Mean + SD (Range) = 14.19 ± 10.59 (4.0-48) (Disease duration)	11 limb onset<br>5 bulbar onset
**Gomez-Pinedo *et al.* (2014)**	3 patients with ALS	3 control patients undergoing lumbar puncture after headache or epileptic attack for diagnostic purposes	NA	NA	NA	NA
**Yanez *et al.* (2014)**	17 patients with ALS	None	ALS: Mean (Range) = 66.1 (40-77)	NA	ALS: Mean (Range) = 8.94 (4-18) (Disease duration)	12 limb onset<br>5 bulbar onset
**Ding *et al.* (2015)**	18 patients with sporadic ALS<br>8 patients with sporadic ALS and FTD<br>All ALS patients had 'definite' ALS	15 non-neurological control patients (NNC)	ALS: Mean + SD = 47 + 6.2<br>ALS-FTD: Mean + SD = 66 + 8.4<br>NNC: Mean + SD = 54 + 4.2	ALS: 38.9%<br>ALS-FTD: 25%<br>NNC: 33.3%	NA	ALS:<br>15 limb onset<br>3 bulbar onset<br>ALS-FTD:<br>8 limb onset
**Sharma *et al.* (2015)**	10 drug-naïve patients with 'definite' ALS	10 age and gender-matched subjects undergoing spinal anaesthesia for orthopaedic surgery	ALS: Mean + SD = 47.38 ± 5.38	ALS: 20%	ALS: Range = 6-15 (Disease duration)	9 limb onset<br>1 bulbar onset
**Vijayalakshmi *et al.* (2015)**	5 drug-naïve patients with sporadic ALS ('probable' or 'definite')	5 age- and gender-matched neurological control patients (2 with acquired peripheral neuropathy, 2 with idiopathic intracranial hypertension and 1 with normal pressure hydrocephalus)	ALS: Mean + SD (Range) = 56.60 + 6.99 (50-66)	ALS: 20%	ALS: Mean + SD (Range) = 8.2 ± 5.5 (5-18) (Disease duration)	NA

**Galan *et al.* (2017)**	31 patients with ‘probable’ or ‘definite’ ALS	None	ALS: Mean + SD = 59.0 ± 10.1	ALS: 45.2%	ALS: Mean + SD = 30.3 + 24.4 (Survival time)	19 limb onset<br>12 bulbar onset
**Shruthi *et al.* (2017)**	5 drug-naïve patients with ‘probable’ or ‘definite’ ALS	5 age and gender-matched neurological control patients with diseases such as peripheral neuropathy, idiopathic intracranial hypertension and normal pressure hydrocephalus	NA	NA	NA	NA
**Sumitha *et al.* (2019)**	5 patients with sporadic ALS	5 neurological control patients with benign intracranial hypertension<br>(NC)	ALS: Mean + SD (Range) = 58.4 ± 4.3 (52-62)<br>NC: Mean + SD (Range) = 61 ± 6.2 (50-65)	ALS: 40%<br>NC: 40%	ALS: Mean + SD (Range) = 12.6 ± 5.4 (6-18)<br>(Disease duration)	3 limb onset<br>2 bulbar onset
**Tokuda *et al.* (2019)**	10 patients with sporadic ALS<br>1 patient with familial SOD1-ALS	15 neurodegenerative control patients with diseases including Parkinson’s disease, dementia with Lewy bodies and progressive supranuclear palsy (NC)<br>11 non-neurodegenerative control patients with diseases including hypertension, epilepsy, torticollis, cervical dystonia, brainstem infarction, suspected hysteria, muscle atrophy and blepharospasm (NNC)	ALS: Mean + SD = 66.9 ± 11.0*<br>NC: Mean + SD = 68.6 + 8.7<br>NNC: Mean + SD = 58.0 + 13.3**	ALS: 72.7%<br>NC: 26.7%<br>NNC: 63.6%	NA	8 limb onset<br>3 bulbar onset

NA: not available
*Calculated from 10 subjects (Data for 1 subject not available)
**Calculated from 9 subjects (Data for 2 subjects not available)

Supplementary Table S2. Quality assessment

Author/s (Year of publication)	Peer-reviewed publication	Appropriate study approval stated	Appropriate control group identified	Procurement and maintenance of *in vitro* model described	Appropriate description of CSF preservation process	Storage temperature of CSF provided	Blinded assessment of outcome stated	Number of replicates performed stated	Statement of potential conflict of interests
**Askanas *et al.* (1981)**	✓	X	✓	✓	X	X	X	✓	X
**Silani *et al.* (1987)**	✓	X	✓	✓	✓	✓	X	X	X
**Swift *et al.* (1988)**	✓	X	X	✓	X	X	✓	✓	X
**Couratier *et al.* (1993)**	✓	X	✓	✓	X	✓	X	✓	X
**Couratier *et al.* (1994)**	✓	X	✓	✓	X	✓	X	✓	X
**Iwasaki *et al.* (1995)**	✓	X	✓	✓	X	✓	X	✓	X
**Terro *et al.* (1996)**	✓	X	✓	✓	X	X	✓	✓	X
**Smith *et al.* (1998)**	✓	✓	X	✓	✓	✓	✓	✓	X
**Tikka *et al.* (2002)**	✓	✓	✓	✓	✓	✓	✓	✓	X
**Sen *et al.* (2005)**	✓	✓	✓	✓	✓	✓	X	✓	X
**Anneser *et al.* (2006)**	✓	X	✓	✓	✓	X	X	✓	X
**Shobha *et al.* (2007)**	✓	✓	✓	✓	✓	✓	X	✓	X
**Vijayalakshmi *et al.* (2009)**	✓	✓	✓	✓	✓	✓	X	✓	X
**Fiszman *et al.* (2010)**	✓	✓	✓	✓	✓	✓	X	✓	X
**Barber *et al.* (2011)**	✓	✓	✓	✓	✓	✓	X	✓	✓
**Kulshreshtha *et al.* (2011)**	✓	✓	✓	✓	✓	✓	X	✓	X
**Vijayalakshmi *et al.* (2011)**	✓	✓	✓	✓	X	✓	X	✓	X
**Yanez *et al.* (2011)**	✓	✓	✓	✓	✓	✓	✓	✓	X
**Varghese *et al.* (2013)**	✓	✓	✓	✓	✓	✓	X	✓	✓
**Gomez-Pinedo *et al.* (2014)**	✓	X	✓	✓	✓	✓	✓	X	✓
**Yanez *et al.* (2014)**	✓	✓	X	✓	X	X	X	✓	X
**Ding *et al.* (2015)**	✓	✓	✓	✓	✓	X	X	✓	✓
**Sharma *et al.* (2015)**	✓	✓	✓	✓	✓	✓	X	✓	✓
**Vijayalakshmi *et al.* (2015)**	✓	✓	✓	✓	✓	✓	X	✓	✓
**Galan *et al.* (2017)**	✓	✓	X	✓	✓	✓	✓	X	✓
**Shruthi *et al.* (2017)**	✓	✓	✓	✓	✓	✓	X	✓	X

**Sumitha *et al.* (2019)**	✓	✓	✓	✓	X	✓	X	✓	✓
**Tokuda *et al.* (2019)**	✓	✓	✓	✓	✓	✓	X	✓	✓

Supplementary Table S3. Checklist for assessment of CSF cytotoxicity

CHECKLIST ITEM
**Subjects**<br>- Age<br>- Gender<br>- Disease description (e.g. ALS/FTD, sporadic/familial, definite/probable/possible, *C9ORF72* status…)<br>- Disease duration/Survival time/Time of sample collection<br>- Disease severity/Disease staging (e.g. ALS-FRS score)<br>- Site of onset (bulbar onset or limb onset)<br>- Sample size<br>- Control group
**Culture model**<br>- Type (e.g. human iPSC-derived spinal motor neurons)<br>- Procurement<br>- Maintenance
**CSF handling**<br>- CSF preservation process<br>- Storage temperature and duration
**CSF exposure**<br>- Concentration (v/v%)<br>- Exposure time<br>- Diluent<br>- Serum (presence of serum or serum-free conditions)
**Outcomes**<br>- Outcome/s assessed (e.g. cell viability, apoptosis…)<br>- Assay/s employed (e.g. MTT, LDH…)
**Results**<br>- Outcome measure/s (e.g. cell count, LDH activity…)*<br>- Summary statistic (e.g. mean difference)**<br>- Statistical analysis (statistical test, p-value & significance level)
**Additional items**<br>- Number of replicates performed<br>- Blinded assessment of outcome

*Both pre- and post-CSF exposure values should be provided.

**Study groups involved in calculation of summary statistic should be clearly stated.
